# Adolescents living with HIV in the Copperbelt Province of Zambia: Their reproductive health needs and experiences

**DOI:** 10.1371/journal.pone.0197853

**Published:** 2018-06-05

**Authors:** Donna R. McCarraher, Catherine Packer, Sarah Mercer, Alexis Dennis, Harry Banda, Namakau Nyambe, Randy M. Stalter, Jonathan K. Mwansa, Patrick Katayamoyo, Julie A. Denison

**Affiliations:** 1 FHI, Durham, NC, United States of America; 2 Department of Sociology, University of North Carolina at Chapel Hill, Chapel Hill, NC, United States of America; 3 FHI, Ndola, Zambia; 4 FHI, Lusaka, Zambia; 5 Department of Epidemiology at the University of Washington, Seattle, Washington, United States of America; 6 Arthur Davison Children’s Hospital, Ndola, Zambia; 7 Department of International Health, Johns Hopkins Bloomberg School of Public Health, Baltimore, MD, United States of America; Brown University, UNITED STATES

## Abstract

**Background:**

Understanding and meeting the reproductive health needs of adolescents living with HIV (ALHIV) is a growing concern since advances in antiretroviral therapy mean that many ALHIV are now living into adulthood and starting to have sex.

**Methods:**

We conducted a mixed-methods study in the Copperbelt Province of Zambia to advance our understanding of the reproductive health needs of ALHIV and to assess the extent to which these needs are being met. We conducted in-depth interviews (IDIs) with 32 ALHIV from two HIV clinics, 23 with their caregivers, and 10 with clinic staff. ALHIV were interviewed twice. We used the data from the qualitative interviews to create a cross-sectional survey that we conducted with 312 ALHIV in three HIV clinics.

**Findings:**

The vast majority of ALHIV reported they wanted to have children in the future but lacked knowledge about preventing mother-to-child transmission. Some sexually active adolescents used condoms, although they wanted more information about and access to non-condom methods. Many ALHIV reported that their first sexual encounters were forced. Religious beliefs prevented some caregivers from discussing premarital sex and contraception with ALHIV. Clinic staff and caregivers had mixed views about integrating contraceptive counseling and method provision into HIV care and treatment services. Few sexually active ALHIV reported that they disclosed their HIV status to their sexual partners and few reported that they knew their sexual partner’s status.

**Conclusions:**

ALHIV are in dire need of comprehensive sexual and reproductive health services and information including a range of contraceptive methods to prevent pregnancy, knowledge about preventing mother-to-child transmission and having a healthy pregnancy, skills related to HIV disclosure and condom negotiation to prevent horizontal transmission, and screening for sexual violence for both males and females if services are available.

## Introduction

Advances in and improved access to antiretroviral therapy (ART) have resulted in HIV becoming a chronic illness with children born with HIV living into adolescence and adulthood. Globally, young people ages 15–24 account for 34% of all new HIV infections[1] and represent a growing population in need of HIV care and treatment. HIV care and treatment programs, however, are typically designed to serve infants, children, and adults and few are equipped to address the needs of adolescents. Meeting the needs of adolescents living with HIV (ALHIV) ages 15–19 is critical since by this age about 25% of adolescents many adolescents in sub-Saharan Africa, where most ALHIV live, have begun childbearing[2]. As such, this age group needs information and access to contraceptive methods and secondary prevention strategies to prevent re-infecting themselves and transmitting the HIV virus to their children and sexual partners [3, 4].

There is a paucity of data on the sexual and reproductive health (SRH) needs of ALHIV. We found seven studies with data from sub-Saharan Africa on this topic that were conducted in Kenya [5], Uganda [6, 7], Tanzania [8], Zambia [9, 10], and the Democratic Republic of Congo (DRC)[11]). A cross-sectional survey from Kenya [5] reported high rates of unintended pregnancy and repeated unintended pregnancies. A mixed-methods study in Uganda reported high rates of condom use among sexually active ALHIV but focus group discussions suggest that condom use was inconsistent [6]. Qualitative studies highlight contextual factors that inhibit the ability of ALHIV to engage in safe sex practices. Research conducted in Tanzania [8] and Zambia [10]) reported that adolescents were reticent to disclose their HIV status to their sexual partners because they were afraid of being rejected. These studies also found that strong cultural norms restricted conversations between the adolescents and their caregivers on SRH issues. Health care workers in Zambia reported that long queues, under-staffing at the clinics, and high work load made it difficult to have private, high-quality counseling sessions with ALHIV [9]). In the DRC, providers were uncomfortable discussing sex and providing condoms to HIV-positive young people and were confused about whether it was legal to provide family planning counseling to youth under the age of 18 [11]). In sum, these articles suggest that ALHIV ages 15–19 are at risk for unintended pregnancy; transmission of the virus to their sexual partners and children; and superinfection, whereby an HIV-infected person acquires a different strain of HIV from another HIV-infected person.

In this paper, we present the results from a mixed-methods study that was conducted to inform programming for ALHIV in Zambia. Findings from this study have been published previously include perspectives from ALHIV on living with HIV and HIV self-management, an analysis of factors related to incomplete ART adherence, and characteristics of ALHIV who are on second-line ART treatment [12–14]. In this paper, we focus solely on the SRH needs of ALHIV from multiple perspectives, including adolescents themselves, their caregivers, and clinic providers. This study expands the current knowledge base on the understudied SRH needs of ALHIV.

## Materials and methods

### Study overview

From 2011 to 2013, we conducted in-depth interviews (IDIs) and a cross-sectional survey in three HIV clinics in the Copperbelt Province: a children’s hospital (Clinic 1) and two central hospitals (Clinics 2 and 3). All clinics offered adolescent-specific clinic days and two provided monthly adolescent support group sessions. This study was reviewed and approved by FHI 360’s Protection of Human Subjects Committee, the Eres Converge Institutional Review Board in Zambia, and the Zambia Ministry of Health.

#### Qualitative phase

We sought to conduct IDIs with 16 male and 16 female ALHIV (ages 15–18) who were on ART, their caregivers, and all clinic staff in the children’s hospital and in one of the central hospitals.

To be eligible to participate in an IDI, the adolescents had to be aware of their HIV status, taking ART, and willing to be interviewed twice. We selected adolescent clients to have equal numbers of males and females and equal numbers of adolescents ages 15–18 at each clinic. Initially, clinic staff selected adolescents to participate in the IDIs, but the study team became concerned that only patients emotionally close to the providers, or those who may be highly adherent to their ART regime, would be invited to participate. To capture a variety of experiences, the team randomly selected youth attending the clinic on a given day until 16 males and 16 females were enrolled.

Adolescent participants were invited to complete two IDIs, that each lasted about one hour. The first interview focused on the participants’ experiences with HIV care and treatment, including adherence to ART, and the second interview focused on the participants’ SRH needs (Table 1). We conducted two separate IDIs with ALHIV to build rapport between the interviewers and the ALHIV. We felt this was necessary to help participants feel more comfortable with discussing matters related to sex. The IDIs were conducted to provide contextual information on ALHIVs’ experiences related to HIV and SRH and to develop relevant and well-informed questions for the quantitative survey.

**Table 1:** Domains explored with the IDIs related to sexual and reproductive health.

ALHIV	Caregivers	Clinic staff
• Experiences in intimate relationships and sharing HIV status with partners • Experiences with sex, pregnancy, HIV prevention, and condom use • Desires for future children • Where they get information from and what future information they want	• Views on sexual behavior among adolescents • Thoughts on what adolescents should know about sex • Who should provide adolescents with information on sex • What would help adolescents practice safe sex • Thoughts on providing family planning (FP) counseling and methods at the ART clinic	• Views about FP and pregnancy prevention among adolescents • What information do adolescents need about sex and FP and where do they get it • Thoughts on providing FP counseling and methods at the ART clinic

With the adolescents’ permission, their adult caregivers were invited to participate in an IDI. Caregivers were defined as adults over the age of 19 who knew the adolescents’ HIV status, helped care for them, and were the adult contact person for the clinic. We invited all clinic staff who worked at the clinic, interacted with ALHIV, and were available to participate in an IDI. Written consent was obtained before the IDIs were conducted. We obtained a waiver of parental consent for ALHIV between the ages of 15 and 17, since young people can access ART services without the consent of their parent or guardian starting at age 12, and because we felt that requiring parental or guardian consent might bias study participation. ALHIV and their caregivers who participated in the IDIs were reimbursed about US$6 to pay for their travel to the clinic and their time. Interviews were conducted in either Bemba, Nyanja, or English, and were audio recorded with the participants’ permission.

#### Quantitative phase

We used the data from the IDIs from the first phase to develop a quantitative survey to generate data to inform future strategies to support ALHIV. Trained interviewers used personal digital assistants (PDAs) to conduct the survey. All adolescents between the ages of 15 and 19, who had been attending the ART clinics at the study clinics for at least three months, and were aware of their HIV status, were invited to participate in a one-time survey. We expanded the age range to include ALHIV who were 19 years to increase the number of potential study participants. This age range also reflections the World Health Organization’s definition of older adolescents. Clinic staff introduced the study to eligible ALHIV and referred interested adolescents to a study team member who led the potential participant through the informed consent process. The surveys took place in a private location in the study clinic and took approximately one hour to complete.

From December 2012 to May 2013, we conducted surveys with 312 of the estimated 365 15- to 19-year-old ALHIV attending the HIV clinics. Two ALHIV who were invited declined to participate in the study. Complete data for 309 eligible participants were analyzed. We obtained written consent from the survey participants and paid them about US$10 for their time and travel. As with the IDIs, parental consent for participants ages 15–17 was waived. The survey included questions about forced sex since this topic was spontaneously mentioned in the IDIs. For those participants who reported forced sex experiences, interviewers were trained to refer them to the nurse-in-charge and give them contact information to the sexual- and gender-based violence (SGBV) unit at one of the study hospitals and the number to an SGBV hotline.

### SRH survey measures

We asked adolescents if they currently had a boyfriend or girlfriend, if they had ever had vaginal sex, if they had had sex more than one time, if they had sex in the past 12 months and since learning their HIV status, age at first sex, age and HIV status of first and last sexual partner, whether they used anything to prevent pregnancy the first and last time they had sex, and whether their first and last sexual partner knew their HIV status. We asked participants if their first sex was forced—the specific question was, “The first time you had sex, did you do it because you wanted to or because you were forced to do it against your will?” Other SRH-related survey questions asked about ever being pregnant or making a female pregnant, whether they planned to use a contraceptive method in the future, and desires to have children in the future. SRH service-related questions asked about their comfort talking to clinic staff about pregnancy prevention and sex, whether clinic staff have ever offered them condoms or demonstrated condom use, and what topics related to HIV information they would like more information about.

### Data management and analysis

All IDIs were transcribed verbatim and translated (when necessary) into English, reviewed for accuracy, and entered into NVivo v.8 (QSR International). We used an iterative process to analyze the data with initial codebooks developed for each participant type based on the IDI guides. To ensure intercoder reliability, 15% of the transcripts were coded by at least two of the authors (HB, CP, and JAD) with discrepancies resolved through discussion. As the analysis progressed, and the team identified new themes and discussed coding discrepancies, the codebooks were revised accordingly. Coding reports were generated from NVivo and matrices were developed for each participant type summarizing the main themes of each coding report.

The quantitative data collected using PDAs were uploaded weekly on a password-protected laptop and sent via a secure server to the study manager. The study manager reviewed the data weekly and queries about data discrepancies were sent to the study team in the field for resolution. We used SAS software (version 9.3) to analyze the quantitative data. We conducted descriptive analyses, stratifying the data by sex. The analyses presented in Tables 2 through 4 were conducted separately by two research analysts to verify that the data presented were correct.

**Table 2:** Demographic characteristics of study participants stratified by sex.

	Males (N = 147)	Females (N = 162)	Total (N = 309)
Client characteristics	n (%)	n (%)	n (%)
**Age**			
15	25 (17.0)	23 (14.2)	48 (15.5)
16	40 (27.2)	40 (24.7)	80 (25.9)
17	36 (24.5)	35 (21.6)	71 (23.0)
18	28 (19.0)	32 (19.8)	60 (19.4)
19	18 (12.2)	32 (19.8)	50 (16.2)
Mean (SD)	16.8 (1.3)	17.1 (1.3)	16.9 (1.3)
**Clinic**			
Clinic 1	65 (44.2)	77 (47.5)	142 (46.0)
Clinic 2	63 (42.9)	52 (32.1)	115 (37.2)
Clinic 3	19 (12.9)	33 (20.4)	52 (16.8)
**Currently enrolled in school**	125 (85.0)	117 (72.2)	242 (78.3)
**Biological parents**			
Neither alive	46 (31.3)	66 (40.7)	112 (36.2)
Only father alive	23 (15.6)	24 (14.8)	47 (15.2)
Only mother alive	44 (29.9)	33 (20.4)	77 (24.9)
Both parents alive	33 (22.4)	39 (24.1)	72 (23.3)
Missing	1 (0.7)	-	1 (0.3)
**Current living situation**			
Lives with a biological parent	84 (57.1)	78 (48.1)	162 (52.4)
Lives with other family members not parents	58 (39.5)	77 (47.5)	135 (43.7)
Lives at orphanage	5 (3.4)	7 (4.3)	12 (3.9)
**Lives with others who have HIV**	54 (36.7)	61 (37.7)	115 (37.2)
**How acquired HIV**			
Perinatally infected	113 (76.9)	125 (77.2)	238 (77.0)
Unprotected consensual sex	1 (0.7)	6 (3.7)	7 (2.3)
Rape/forced sex	-	6 (3.7)	6 (1.9)
Contaminated blood	3 (2.0)	3 (1.9)	6 (1.9)
Shared injection	3 (2.0)	5 (3.1)	8 (2.6)
Don't know	27 (18.4)	17 (10.5)	44 (14.2)
**Age learned HIV status**			
Mean (SD)	12.0 (2.4)	12.7 (2.8)	12.3 (2.7)
Median (Range)	12.0 (5.0 to 18.0)	13.0 (4.0 to 19.0)	13.0 (4.0 to 19.0)

**Table 3:** Sexual and reproductive health characteristics of study participants stratified by sex.

	Males (N = 147)	Females (N = 162)	Total (N = 309)
**Has boyfriend/girlfriend**	37 (25.2%)	60 (37.0%)	97 (31.4%)
**Ever had sex**	29.0 (19.7%)	35 (21.6%)	64 (20.7%)
			
	(N = 29)	(N = 35)	(N = 64)
**Had sex only one time**	13 (44.8%)	17 (48.6%)	30 (46.9%)
**Had sex past 12 months**	18 (62.1%)	23 (67.6%)	41 (65.1%)
**Had sex since learning HIV status**	22 (75.9%)	26 (74.3%)	48 (75.0%)
**Age at first sex**			
Mean (SD)	14.7 (2.5)	15.1 (3.0)	14.9 (2.8)
Median (range)	15.0 (10.0 to 19.0)	16.0 (7.0 to 18.0)	16.0 (7.0 to 19.0)
**First sex forced**	9 (31.0%)	22 (62.9%)	31 (48.4%)
**Age of first sex partner**			
Mean age	15.5 (3.2)	23.6 (9.9)	19.7 (8.4)
Median (range)	15.0 (11.0 to 25.0)	21.0 (10.0 to 65.0)	18.0 (10.0 to 65.0)
**First partner knew HIV status**	3 (10.3%)	4 (11.4%)	7 (10.9%)
**Knew HIV status of first partner**	3 (10.3%)	14 (40.0%)	17 (26.6%)
**Used contraceptive method at first sex**	13 (44.8%)	21 (58.3%)	34 (52.3%)
**Ever been pregnant**	-	2 (5.7%)	2 (3.1%)
**Ever made female pregnant**	1 (3.5%)	-	1 (1.6%)
	**(N = 16)**	**(N = 18)**	**(N = 34)**
**Age of last sex partner**			
Mean age	15.8 (2.3)	24.2 (6.9)	20.1 (6.7)
Median (range)	15.5 (12 to 20)	23 (18 to 45)	19 (12 to 45)
**Last partner knew HIV status**	2 (12.5%)	7 (40.0%)	9 (26.5%)
**Knew HIV status of Last partner**	2 (12.5%)	7 (40.0%)	9 (26.5%)
**Used contraceptive method at last sex**	6 (37.5%)	11 (61.1%)	17 (50.0%)
	**(N = 147)**	**(N = 162)**	**(N = 309)**
**Plan to use contraceptive method in the future**	104 (70.8)	100 (61.7)	204 (66.0)
**Want child in future**			
Does not want children	6 (4.1%)	0	6 (1.9%)
Wants children now	0	1 (0.6%)	1 (0.3%)
Wants children in the future	141 (95.9%)	161 (99.4%)	302 (97.7%)

**Table 4:** SRH clinic experiences and information needs by sex.

	Males (N = 147)	Females (N = 162)	Total (N = 309)
	n (%)	n (%)	n (%)
**Comfortable talking to clinic staff about pregnancy prevention**	108 (73.5)	144 (88.9)	252 (81.6)
**Talked with clinic staff about sex**	92 (62.6)	105 (64.8)	197 (63.8)
**Clinic staff offered condoms**	25 (17.0)	15 (9.3)	40 (12.9)
**Clinic staff demonstrated condom use**	41 (27.9)	37 (22.8)	78 (25.2)
**Would like more information on:**			
Gaining skills/knowledge to get a job	141 (95.9)	158 (97.5)	299 (96.8)
Having a healthy baby	137 (93.2)	151 (93.2)	288 (93.2)
Not infecting a sexual partner	138 (93.9)	147 (90.7)	285 (92.2)
How to deal with stigma/prejudice	125 (85.0)	144 (88.9)	269 (87.1)
Adherence to ART	118 (80.27)	135 (83.3)	253 (81.9)
Family planning	110 (74.8)	111 (68.5)	221 (71.5)
HIV disclosure	108 (73.5)	126 (77.8)	234 (75.7)
Relationships (boyfriend/girlfriend)	111 (75.5)	124 (76.5)	235 (76.1)
Other	5 (3.4)	4 (2.5)	9 (3.0)

## Results

### Description of qualitative participants

We conducted initial IDIs with 32 ALHIV (16 males and 16 females), and 31 of them completed a second IDI, which focused on SRH. We interviewed a total of 23 caregivers; 19 were female (aunts, mothers, sisters, grandmothers, and a stepmother) and four were male (uncles, a father, and a cousin). Nine of the caregivers invited to participate in the study refused because they were too busy, had to work, lived far away, or did not want to be seen at an HIV clinic. We conducted IDIs with 10 clinic staff, who included adherence counselors, nurses, data entry clerks, a clinical officer, and a pharmacy technologist. We did not originally intend to interview the HIV clinic data clerks. However, as the data collection began we found that in general the data clerks were younger than the other staff and that they provided the ALHIV with their test results. As such, the data clerks were a source of information and support to the ALHIV and, therefore, we decided to include them in the IDIs. Seven of the 10 clinic staff interviewed were female.

### Sociodemographic characteristics of adolescent survey participants

Forty-eight percent of the study participants were male and 52% were female (Table 2). The mean age of the survey participants was 16.9 years. About half of those interviewed were from the Children’s Hospital, Clinic 1. Almost 80% of those interviewed were currently enrolled in school, with more males in school (85%) than females (72.2%). Slightly over a third (36.2%) reported that neither of their biological parents were living. The majority lived either with a biological parent (52.4%) or another family member (43.7%). Over a third reported that they lived with others who have HIV. Slightly more than three-quarters of participants self-reported that they were perinatally infected.

### Sexual experiences including forced sex

In the survey, more females (37%) than males (25.2%) reported that they currently had a boyfriend or a girlfriend (Table 3), and a fifth of participants reported they had ever had sex. Of those who had sex, almost half (46.9%) reported that they had only had sex once. The mean age at first sex was 14.9 years.

Reported experiences of nonconsensual sex were high: of those who had ever had sex, 31% of males and 62.9% of females reported in the survey that their first sexual encounter was forced. Four adolescents in the IDIs (three females, one male) discussed experiences of forced sex, three of whom reported that the forced sex was their only experience with sex. In the IDIs, participants reported that perpetrators were family members (step-father, uncle, sister-in-law), and a male friend. All four who were raped by a family member reported that this was how they acquired HIV. One male IDI participant and one female IDI participant were afraid that disclosing the violence would lead to them being evicted from the home. A female (age 18) said, *“My step-father is the one who raped me…when I went to the clinic and then they told me that you have got HIV…I told my mum and then she wanted to go to the police and then he threatened my mum*.*”*

A few caregivers said that some of the adolescents’ sexual experiences stemmed from sexual abuse and felt that ALHIV need to be educated to avoid situations that make them vulnerable to violence.

Specific strategies that were mentioned included not letting young children sit on adult male laps, not letting males and females share the same sleep quarters, and encouraging young girls to say “no” firmly to inappropriate touch. As stated by this grandmother of a 17-year-old female, *“What can I emphasize on these children of ours should be scared of men when they are playing and he touches a breast…To be firm for herself as a young girl she should be able to say no*.*”*

### Sex partner age and HIV disclosure

In the survey, females reported that their first sexual partner was older than they were, whereas males reported that their first sexual partner was about the same age. Only about 10% reported that their first partner knew their HIV status, but more females than males reported that they knew their first partner’s HIV status (40% vs. 10.3%). The age of last sexual partner and knowledge of last sexual partner’s HIV status were similar to those reported for first sexual partners. Similarly, in the qualitative IDIs, reports of HIV disclosure to sex partners was rare, mostly because of fear of gossip or rejection by partners. A 17-year-old female explained why she had not disclosed her HIV status to her sex partner:

“Ok we’ve never discussed it because I don’t really know if he’s going to be there for me or what I will tell my partner when the right time comes, because sometimes you may tell people you are HIV positive and they go on spreading rumors about you something that you don’t want, so that’s how it is.”

### Contraceptive use

Fifty percent of the surveyed ALHIV who reported having had sex said that they used a contraceptive method at last sex. Contraceptive use at last sex was higher among females (61.1%) than males (37.5%) (Table 3). The vast majority of sexually active ALHIV who reported using contraceptive methods at last sex used male condoms as opposed to other contraceptive methods (data not shown). IDI participants gave several reasons for not using contraception, including having only infrequent or spontaneous sex. In addition, some youth had misperceptions about pregnancy and pregnancy prevention, as illustrated by this male, age 18, *“Because then we were young and not yet grown-ups…Because we thought maybe young people don’t get pregnant*.*”*

### Fertility desires and prevention of mother-to-child transmission of HIV

Ninety-three percent of ALHIV surveyed wanted more information on how to have children without infecting their baby or their partner (Table 4). Most qualitative IDI participants were asked about their understanding of prevention of mother-to-child transmission (PMTCT) of HIV. Most were aware of the concept of “PMTCT” and knew that there are ways to avert vertical transmission of the virus from the mother to child. Some of the qualitative IDI participants understood that pregnant women could take ART and should avoid or limit breastfeeding, and two participants were aware that a cesarean delivery can reduce HIV risk. However, knowledge about specific PMTCT methods was far from comprehensive. One 18-year-old female said, *“I don’t know what really happens how the baby catches the virus so I don’t really know what exactly just know that through giving birth maybe there is blood*.*“*

Survey participants also had gaps in their knowledge of PMTCT; for example, when asked if clinic staff had ever discussed specific ways of having a healthy baby, less than a quarter said clinic staff had talked about having a Caesarean section, one-third said they’d talked about giving the baby ART, and less than half said they’d talked about the mother taking ART or exclusive breastfeeding (data not shown).

Almost all adolescent participants in the IDIs and survey reported they wanted children in the future (Table 3). Most participants reported they wanted to wait to have children until after they finished school, after they were married, when they had a job, and/or when they had income (data not shown). One 18-year-old female IDI participant described wanting children in the future and how she felt hopeful that she could have an HIV-negative baby:

“I would really like to have children and I really want to do that but, that is coming maybe after I graduate yah, I finish my school, and yah I know maybe I will get married in the same situation that I am in. But thank God that they have brought this thing that testing what is voluntary when you are pregnant and so they can prevent the baby from getting the disease so that’s the good thing. Yah it really motivates me, yah, so I would love to have children.”

### Interactions with clinic staff about SRH

Adolescent IDI participants held overall positive views of the clinic staff in the HIV clinics they attended; they commented that the clinic staff were “kind” and “caring” and that they felt at ease talking with them in general. However, very few discussed their interactions with clinic staff about their SRH needs. In the survey, over 80% of adolescents reported they would feel comfortable talking to the clinic staff about preventing pregnancy (Table 4) (female = 88.9%, male = 73.5%). And more than three-quarters of adolescents reported wanting more information about having an HIV-negative baby, about how to not infect a sexual partner, and about contraception (Table 4).

Male condoms are available for free at the HIV clinics and some clinic staff who completed IDIs reported giving condoms to ALHIV at the clinic. When asked about this in the survey, only 12.9% of ALHIV reported they were offered condoms by a clinic staff; almost twice the number of males reported they were offered condoms as females (17.0% vs. 9.3%). Only about a quarter reported that a clinic staff person had demonstrated condom use to them. In addition, while a few clinic staff reported that they referred ALHIV to family planning clinics elsewhere for non-condom methods, only one adolescent from the survey reported being referred to a family planning clinic (data not shown).

### Clinic staff and caregiver’s perceptions on providing contraceptive services as part of HIV care

Qualitative data showed that clinic staff and caregivers felt strongly that abstinence should be the primary response to adolescents having sex. Clinic staff felt that adolescents should wait to have sex until they had finished their education and were ready to provide financially and care for a child. For example, one member of the clinic staff said, “*They need to be ready for the children until the time when they are ready to take care of them financially*, *spiritually*, *and morally*. *This is after maybe they finish college or maybe they are working*.*”* Caregivers, on the other hand, brought up religious and moral reasons to abstain. For example, a mother of a 16-year-old male said, *“They should be cautioned all the time to say*, *sex before marriage is wrong*. *I think God’s principles are supposed to be followed*.”

Despite the emphasis on abstinence, both clinic staff and caregivers thought that adolescents should use condoms if they “failed” to abstain. In addition, when asked about how they felt about the prospect of offering contraceptive counseling and methods at the ART clinic, more than half of clinic staff and half of caregivers supported this idea. Caregivers cited the benefits of ALHIV being counseled on ART and contraception at the same time and the need to prevent unintended pregnancy. Clinic staff requested additional training to counsel on non-condom methods and said that they would likely be the appropriate people to counsel ALHIV on contraception. For example, *“these children they are able to express themselves with us…we know them properly if they know us they are free to talk to us in any way…to talk to us if they have a boyfriend*, *they want to try something…even there it could be the best way of telling them information on those things [contraception]*.*”*

While many supported the idea of providing contraceptive services to ALHIV at the ART clinics, some caregivers and clinic staff had either mixed or negative feelings about this. Caregivers’ opposition mainly stemmed from the concern that counseling and offering contraception to ALHIV would encourage them to have sex. As stated by an aunt of a 16-year-old female, *“You will encourage now saying that now there is family planning you can go and have sex among yourselves* … *we will protect you against pregnancies*.” In addition, some clinic staff had concerns that promoting contraceptive methods other than condoms would lead to non-use of condoms. For instance, *“Mostly*, *I ask them to use condoms because if I tell them to use this pill or the injectable it will mean they will be re-infecting each other*, *but protecting from pregnancy*. *“*

## Discussion

Our study population had multifaceted SRH needs. While only a fifth of survey participants reported that they had had sex, almost all ALHIV desired children in the future. Knowledge and use of contraception was limited to mainly condom use, and still, only half of those who had sex reported that they used a condom at last sex. Taken together, these finding suggest that our study participants need contraceptive counseling and method provision to prevent unintended pregnancies. In addition, while participants were knowledgeable about PMTCT in general, few could articulate specific strategies. To optimize maternal and infant health, ALHIV need to be better informed on PMTCT strategies. This need is especially important for ALHIV, given that by age 19, 50% of youth in Zambia are currently pregnant or have had a child [15]. Finally, a sizable proportion of male (31%) and female survey (63%) participants reported that their first sex was forced. Some adolescents reported that forced sex was the way in which they acquired HIV. Unfortunately, caregivers’ strategies to prevent sexual violence were inadequate; however, they supported providing adolescents (and children) with information and strategies to prevent their victimization.

Our findings both echo and deviate from previous work. For example, the participants in this study population reported being less sexually active than studies of ALHIV in Kenya [5] and Uganda [16] but similar to another study conducted in Zambia [17]. Consistent with what researchers found in Uganda [6], condoms were the most frequently used method of contraception. Our findings that providers clearly prefer to promote abstinence, followed by condoms, and then other family planning methods were similar to results of studies conducted in Tanzania [8] and the DRC [11]. Both in our study and the DRC study, providers’ reluctance to talk about sex and counsel ALHIV on pregnancy prevention and contraceptive options was influenced by cultural norms that dictate that sex and use of contraception are only appropriate in the context of marriage. Lack of clear service delivery guidelines in both our study and the DRC study were also cited by providers as a reason for not counseling ALHIV on pregnancy prevention and contraceptive methods.

Our study findings highlight that more attention needs to be given to the sexual violence experienced by ALHIV. This study is one of the first to present information on forced sex among ALHIV, ages 15–19 years of age. We found two studies that presented information on ALHIV experiences of violence. In a survey of ALHIV ages 12–18 in Malawi to examine factors associated with depression, Kim and colleagues reported that of 562 participants interviewed 15 percent had experienced forced sex, physical abuse, or had witnessed physical violence in the home [18]. On the other hand, a cross-sectional survey of youth ages 12 to 24 years in Moshi, Tanzania to evaluate their mental health needs, reported that among participants who had mental health difficulties, few reported experiencing sexual abuse [19]. The need to address sexual violence in the fight against HIV has been widely recognized by the global community. In 2013, guidelines for the clinical management of children and adolescents who have experienced sexual violence were developed for the U.S. President’s Emergency Plan for AIDS Relief programs in response to large percentages of patients under the age of 18 presenting for care when sexual assault services were introduced to primary health care centers [20]. The focus of these guidelines is to prevent HIV acquisition through provision of post-exposure prophylaxis.

Our data suggest that ALHIV are also in need of comprehensive sexual violence services, which include not only clinical treatment but also psychological support. This support is especially important since some studies have documented the relationship between experiencing sexual violence and risky sexual behavior among ALHIV [21]. Additionally, associations between sexual violence, HIV, and depression have been documented in the literature [22], and this is gaining attention as a barrier to ART adherence [23, 24]. While screening for violence among ALHIV enrolled in HIV care and treatment is potentially a first step, this should only be implemented if comprehensive services are available on-site or if referrals can be made to existing programs.

There were some limitations to our study. Desirability bias is a threat to the internal validity of our findings. Given that premarital sex was largely considered inappropriate by many in our sample (by caregivers, clinic staff, and some adolescents themselves), underreporting of sexual activity is possible. Other methods of data collection, such as audio computer-assisted self-interviewing, which maximizes confidentially, could increase honest responses about sensitive topics by adolescents. Also, we can only draw conclusions from the ALHIV, caregivers, and clinic staff that we could interview. The SRH needs of those ALHIV whom we did not interview may differ from those of our study population. In addition, our study clinics serve primarily urban populations and our sample consisted of mainly school-going youth. Therefore, experiences of rural or non-school-going youth are not represented.

ALHIV in Zambia are in dire need of comprehensive SRH information. As a first step, the role of providing SRH services to ALHIV within the HIV care and treatment setting must be clearly articulated. This would include clear policies and standards of care on providing SRH information to ALHIV, training providers, and supplying commodities. If SRH services are to be integrated into adolescent HIV care and treatment services, values clarification exercises would help to enable providers to differentiate their own personal beliefs and attitudes—about having sex, people living with HIV having children, and the use of contraception—from the needs of AHLIV.

Given the lack of communication about SRH between ALHIV and clinic staff in our study, additional efforts are needed to improve patient-provider communication. The use of mobile technology to deliver SRH information should also be explored to allow ALHIV to access unbiased information, a strategy that has been adopted to provide young people accurate SRH information in some African countries [25–27]. The SRH needs of ALHIV are not isolated but rather need to be more closely tied to their HIV care and treatment goals. For example, ALHIVs’ near universal desire to have children in the future could be a way to motivate them to adhere to ART, so counseling messages and strategies linking the desire for children to ART adherence should be developed and tested. Finally, integration of SRH services into HIV care and treatment services has largely focused on preventing unintended pregnancies, with much less emphasis on how to have an HIV-negative baby. As safe conception guidelines and tools for people living with HIV are rolled out in resource-poor settings, the needs of ALHIV must be considered.
